# A nomogram model based on the combination of the systemic immune-inflammation index, body mass index, and neutrophil/lymphocyte ratio to predict the risk of preoperative deep venous thrombosis in elderly patients with intertrochanteric femoral fracture: a retrospective cohort study

**DOI:** 10.1186/s13018-023-03966-4

**Published:** 2023-08-03

**Authors:** Guowei Zeng, Xu Li, Wencai Li, Zhijia Wen, Shenjie Wang, Shaowei Zheng, Xia Lin, Haobo Zhong, Jianping Zheng, Chunhan Sun

**Affiliations:** 1 Present Address: Department of the Orthopedics, Huizhou First Hospital, Guangdong Medical University, Huizhou, 516000 Guangdong China; 2 Present Address: Guangdong Medical University, Zhanjiang, 524000 Guangdong China; 3 Present Address: Department of Neurosurgery, Huizhou Central People’s Hospital, Huizhou, China

**Keywords:** Deep venous thrombosis (DVT), Immune inflammatory factors, Intertrochanter femoral fracture, Nomogram, SII, NLR, BMI

## Abstract

**Objectives:**

Deep vein thrombosis (DVT) has been considered as a frequent and serious consequence of intertrochanteric femoral fractures in the elderly. Several negative repercussions of DVT can be considerably mitigated by its timely recognition and treatment. The current work was aimed at exploring the factors independently predicting DVT among cases suffering from intertrochanteric femoral fractures and validate their predictive usefulness in diagnosing DVT.

**Methods:**

Between April 2017 and July 2022, clinical information from 209 cases showing preoperative DVT for femoral intertrochanteric fractures were retrospectively evaluated. In patients with femoral intertrochanteric fractures, logistic regression analysis with a backward stepwise method was adopted for detecting independent predictors for the diagnosis of preoperative DVT. Using multivariate logistic regression, a nomogram prediction model was developed and verified with the testing group.

**Results:**

According to multivariate logistic regression model, body mass index (BMI) (OR 0.79, 95% CI 0.63–0.99, *P* = 0.042), neutrophil/lymphocyte ratio (NLR) (OR 7.29, 95% CI 1.53, 34.64, *P* = 0.0012), and systemic immune-inflammation index (SII) (OR 6.61, 95% CI 2.35, 18.59, *P* = 0.001) were independent predictors for DVT before surgery among cases developing intertrochanteric femoral fracture. AUC values were 0.862 and 0.767 for training and testing groups, separately, while their mean errors in the calibration curve were 0.027 and 0.038 separately. Decision curve analysis (DCA) curve revealed a high value of clinical application for both groups.

**Conclusion:**

Upon admission, BMI, NLR, and SII are independent predictors of DVT before surgery among cases developing intertrochanteric femoral fractures. Additionally, the nomogram based on the BMI, NLR, and SII can assist clinicians in determining if preventive and symptomatic therapies are required to improve DVT prognosis and reduce its associated mortality.

**Supplementary Information:**

The online version contains supplementary material available at 10.1186/s13018-023-03966-4.

## Introduction

Intertrochanteric femoral fracture is a type of hip fracture that accounts for 63% of all hip fractures. It is the most commonly seen fracture in the elderly, accounting for 10–15% of all fractures in the elderly population [1, 2]. DVT is a primary factor causing global disability and mortality [3, 4], which is a common consequence of an intertrochanteric femoral fracture. Intertrochanteric fractures particularly predispose to DVT owing to its certain unique characteristics, such as the presence of heavy bleeding, the existence of a hypercoagulable state, overall stress reaction, inflammatory response, and forced immobility due to pain [5].

Because of its non-invasive nature and good reproducibility, ultrasound Doppler is most commonly used for diagnosing lower limb DVT [6]. Color Doppler ultrasonography, on the other hand, struggles to produce good image quality in conditions such as a fixed posture, acute limb edema, and extreme discomfort. In clinical practice, color Doppler ultrasonography is not employed frequently for the regular assessment of lower limb fractures or cannot be done on time, resulting in treating patients with thrombosis out of time. In addition, thrombosis is evaluated clinically by several coagulation indicators, like fibrinogen (FIB) levels, D-dimer levels, prothrombin time (PT), thrombin time (TT), activated partial thromboplastin time (APTT), and platelet count (PLT). Among them, D-dimer, FIB, and PLT are commonly used in the clinical evaluation of thrombosis. Plasma D-dimer concentrations are final products generated by plasminase-dependent cross-linked fibrin breakdown, and its large serum levels indicate hypercoagulability and hyperfibrinolysis. Therefore, D-dimer levels are suggestive of DVT with a high sensitivity of more than 95%. However, their specificity is low, at just 20–40% [7–9]. As a result, D-dimer’s efficacy among the old cases suffering from fractures remains debatable [10]. Furthermore, FIB stimulates blood clotting towards the end of the coagulation process, and an increase in PLT count implies hypercoagulability [11]. These indicators can also be elevated in non-fractured individuals, in which case a single signal has limited specificity and a significant false positive rate (FPR) for diagnosing DVT [12].

Apart from those three major factors in the pathophysiology of thrombus formation, namely, stasis or blood flow changes, blood hypercoagulation, and vascular wall injury, inflammatory molecules, and immune cells are now considered to be major contributors to thrombosis. Budnik and Brill detailed immune factors’ effects on DVT pathogenesis in their review [3]. Colling et al. detailed the mechanisms by which the immune and coagulation processes interact with and regulate each other [13]. Branchford et al. suggested that systemic inflammatory responses are related to an elevated risk of DVT. The ensuing platelet activation enhances the pre-thrombotic state and leads to the development of DVT [14]. Restricted blood flow or stasis induces rapid leukocyte recruitment, which leads to the formation of DVT [15]. Neutrophils, monocyte, lymphocytes, and platelets also make a vital impact on the development of DVT [16–18]. Based on these single indicators, corresponding indices including monocyte/lymphocyte ratio (MLR), platelet/lymphocyte ratio (PLR), neutrophil/lymphocyte ratio (NLR), and (platelet × neutrophil)/lymphocyte (systemic immune-inflammation index, SII) are established. Moreover, these indicators have been confirmed to better show the immune inflammatory state of the body [19–22]. These new combination indexes of peripheral blood-derived markers are progressively utilized in orthopedic surgery and its related complications [23–30].

Therefore, the present study aimed to find independent risk factors for thrombosis in intertrochanteric femoral fractures by integrating the risk factors of thrombosis and novel immune inflammatory indices. On this basis, a prediction model was built to assess the DVT event among cases suffering from intertrochanteric femoral fractures simply and effectively.

## Methods

### Patient and public involvement

This study is a retrospective one, with the research questions and assessments being predetermined and not determined by the patient’s priorities, experience, and preferences. Ethics Committee of First People's Hospital of Huizhou reviewed this work and determined that it would not harm the patient or present any potential risk factors. Therefore, no patient involvement or informed consent was required. Moreover, the findings of the study will be reported to the participants and used in clinical trials to observe any potential clinical benefits. The interventions of this study are examinations which are already available to the patient during their hospitalization and do not require any additional cost to the patient.

### Patient selection

In this retrospective study, patients whose intertrochanteric femoral fractures were diagnosed in the orthopedic department of Huizhou First Hospital during April 2017–July 2022 were included. Patients conforming to criteria below were included: (1) cases hospitalized within 5 days (120 h) of disease inception with a definitive radiographic fracture diagnosis, and (2) those aged over > 60 years (a total of 716 patients). Cases conforming to criteria below were eliminated: (1) patients having insufficient information including medical records and preoperative vascular color doppler ultrasonography results (a total of 247 patients); (2) those aged under 60 years (a total of 113 patients); (3) patients with the following conditions: multiple fractures diagnosed at this admission, pathological fracture diagnosed at this admission, previous and recent anticoagulant use, inflammatory diseases, unclear diagnosis of infection at admission, and anti-infective drug use (a total of 147 patients). Ultimately, a sum of 209 patients was incorporated into our research.

The current work was performed following the Helsinki Declaration and was approved by Huizhou First Hospital's ethical committee (2,022,138). Based on the retrospective character of the investigation, no written informed consent was required. With the aim of protecting patients’ privacy, the identifying information of those who took part in this study was rendered anonymous.

### Data extraction

This work gathered basic clinical information from each case, containing their age, sex, fracture site, time of injury to the hospital (damage time, Dtime), diastolic blood pressure (DBP), systolic blood pressure (SBP), temperature (the first measurement on admission), breaths per minute (BPM, the first measurement on admission), pulse (the first measurement on admission), and a medical history of hypertension, diabetes, smoking, and alcoholism. We determined hypertension with the past hypertension history and SBP/DBP of 140/90 mmHg on admission, whereas diabetic with having the past diabetes history, random blood glucose of 11.1 mmol/L on admission and fasting blood glucose (FBG) of 7 mmol/L. Patients' routine blood, hemostatic function, and other serological test results acquired at emergency department or in one day after admission were collected. The normal range for hemoglobin count was between (115–150) × 10^9^/L and (130–175) × 10^9^/L for women and men, respectively, for RBC count, (4.3–5.8) × 10^9^/L, for WBC count, (2–7) × 10^9^/L, for NC count, (2–7) × 10^9^/L, for LYM count, (0.8–3.5) × 10^9^/L, for MNC count, (0.1–0.6) × 10^9^/L, for PLT count, (100–300) × 10^9^/L, for FIB, (1.8–3.5) g/L, for TT, (14–21) s, for PT–INR, (0.85–1.20), for APTT, (22.5–34.0) s, for D-dimer, (0–0.5) mg/L, and PT, (9.8–13.2) s. Values outside the above-mentioned normal ranges were considered to be abnormal. The correlation inflammatory-immune index was evaluated by collecting PLT, NC, LYM, and MNC data during routine blood investigations performed at the emergency department or right after admission. The formulae used were:

NLR = NC/LYM; PLR = PLT/LYM; MLR = MNC/LYM; and SII = PLT × NC/LYM.

### Outcome event

The event of DVT in the elderly with intertrochanteric femoral fracture was considered the outcome event in this study. The positive result was characterized as DVT, whereas the negative outcome was defined as No DVT. “Thrombus” was used as a label to record whether patients with intertrochanteric femoral fractures developed a DVT, which was recorded according to preoprerative color Doppler ultrasonography. The following were the diagnostic criteria for DVT in color Doppler ultrasonography: 1. Abnormal echoes. 2. The vein beneath the ultrasound probe at the fracture site should not have been compressed shut. 3. The venous thrombus section should have shown no clear indication of blood flow. 4. A reduction in the should have occurred afflicted limb’s blood flow and vascular width. A skilled sonographer used color doppler ultrasonography to evaluate blood vessels in the lower extremities and provided a diagnostic report to identify DVT. The bilateral common femoral vein, superficial/deep femoral vein, popliteal vein, anterior/posterior tibial vein, as well as peroneal vein were all tested according to the aim of the present study. Because of the limited clinical relevance of superficial or myenteric veins, the present work did not include superficial vein thrombi.

### Statistical analysis

Before data analysis, we applied R studio version 4.0.3 (The R Foundation for Statistical Computing, Vienna, Austria) to randomly sample the patients into a 7:3 ratio of training group (n = 147) and testing group (n = 62). SPSS 26 (SPSS Inc., Chicago, IL, USA) was adopted in assessing data for normalcy. Normally-distributed continuous variables were represented by mean and standard deviation and compared by t-test, while abnormally-distributed ones were represented by median and quartile and compared by rank sum test. Numeration variables were represented by percentage n (%) for comparisons among groups by chi-square test or Fisher's test when over 1/5 of cells in contingency table had the predicted frequency of less than 5. To determine the comparability of both groups and to guarantee our prediction model viability, we compared the baseline data of the both groups.

We further classified training group into DVT (n = 28) or No DVT group (n = 119) based on the outcome events. Rank sum test, t-test, Fisher’s test and chi-square test were adopted to compare data between two groups. Two sets of violin plots of deference data were created with R studio (version 4.0.3), which included BMI, WBC, NC, LYM, NLR, PLR, and SII. We drew ROC curves for assessing predictive power of the deference data for DVT. Furthermore, based on these cutoff values, high and low groups within each variable were established. In order to determine the variables connected to the outcome event, univariate logistic regression was initially performed, and odds ratios (ORs) together with associated 95% confidence intervals (CIs) were determined. Significant variables (*P* < 0.05) from the univariate analysis were incorporated into multivariate regression, using a backward stepwise strategy to find independent predictors of DVT. *P* < 0.05 indicated statistical significance for the two-sided tests. A nomogram prediction model was built utilizing age and significant variables found in the multivariate regression and displayed using R studio (version 4.0.3). The predictive model nomogram effectiveness was evaluated by ROC curve and area under the curve (AUC). The calibration curve and mean absolute error were employed with the purpose of assessing the nomogram’s goodness of fit. The clinical effects of the nomogram were evaluated based on decision curve analysis (DCA) using the high-risk threshold.

**Fig. 1:**
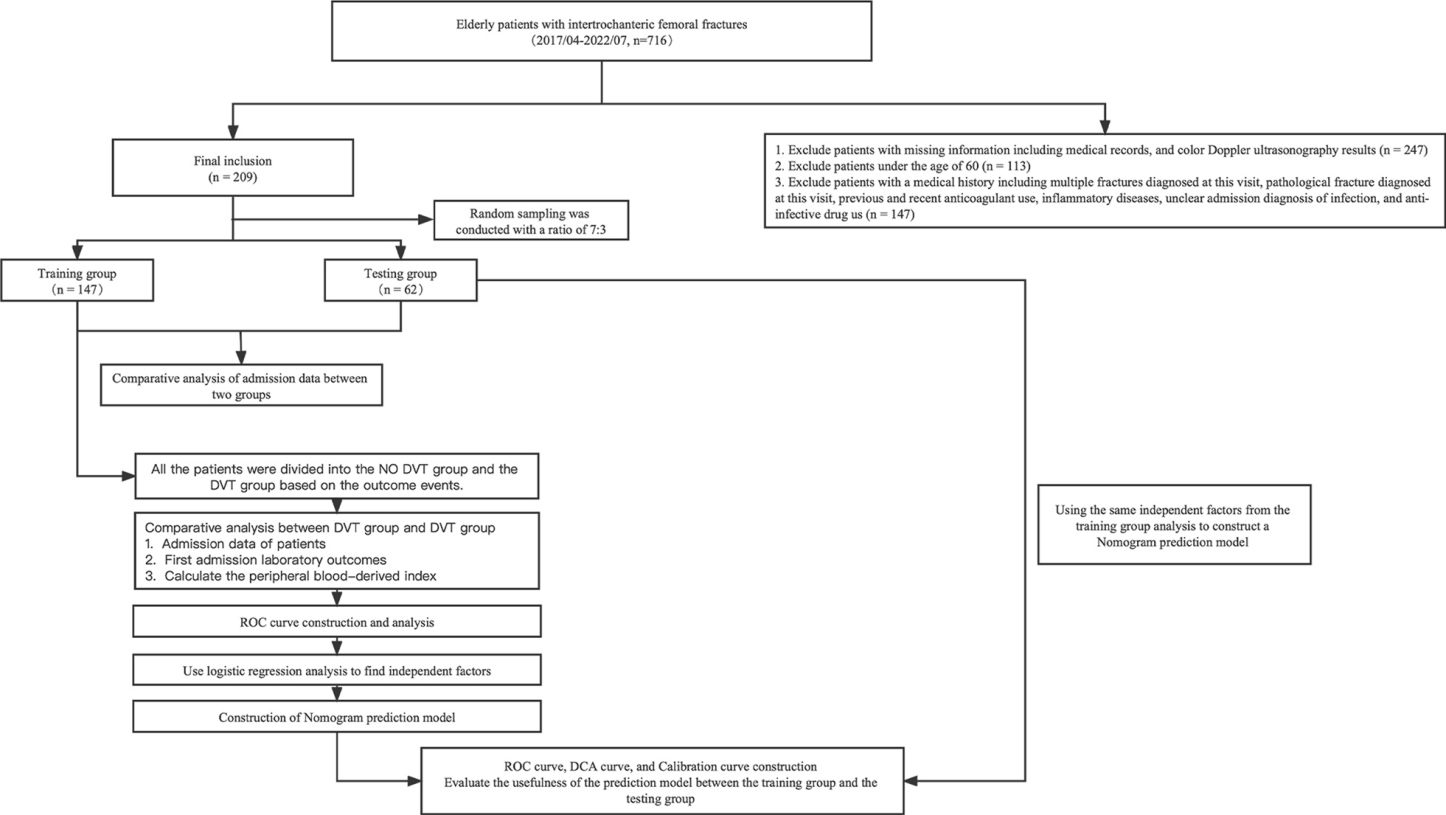
Flow chart. The process of population screening criteria and prediction model establishment in this study

The grouping criteria and model construction process are shown in Fig. 1.

## Results

### Baseline characteristics

The 209 included cases were randomized as training or testing group, in a ratio of 7:3. Thirty-eight men (25.85%) and 109 women (74.15%) comprised the test group with a mean age and BMI of 83 (77, 87) years and 22.43 (21.62, 23.37) kg/m^2^, respectively. Sixteen men (25.81%) and 46 women (74.19%) with a mean BMI of 22.67 kg/m^2^ and age 83.5 (74.5, 87) years, respectively, made up the testing group. All of the variables including sex, age, BMI, alcoholism, smoking, hypertension, diabetes, fracture site, thrombus, DBP, SBP, Dtime, temperature, BPM, and pulse were not significantly different of both groups (*P* > 0.05), supporting the model training and verification on both groups (Additional file 1: Table S1).

Based on the results of preoperative vascular color doppler ultrasonography, the training group was subdivided into No DVT (n = 119) and DVT (n = 28). The No DVT group included 31 men (26.05%) and 88 women (73.95%), their average age was 83 (77, 87) years, while their BMI was 22.43 (21.62, 23.37) kg/m^2^. The DVT group included 7 men (25%) and 21 women (75%), with a mean age of 85 (82, 87.25) years and a BMI of 21.84 (20.7, 22.41) kg/m^2^. BMI was significantly different of both groups (P < 0.05), but no differences in baseline characteristics (*P* > 0.05) such as sex, age, alcoholism, smoking, hypertension, diabetes, fracture site, DBP, SBP, Dtime, temperature, BPM, and pulse. Following admission, a series of routine blood tests (HGB, RBC, WBC, NC, LYM, MNC, and PLT) and tests for coagulation (FIB, TT, PT–INR, APTT, D-D dimer, and PT) were carried out to complete the study data collection. There existed significant differences in WBC, NC, and LYM levels in DVT compared with No DVT groups (*P* < 0.05), while MNC, PLT, FIB, TT, PT–INR, APTT, D-D dimer, and PT levels did not show significant difference (*P* > 0.05). We acquired the correlation inflammatory-immune indices including MLR, NLR, PLR, and SII based on routine blood test indexes; typically, PLR was notably different in DVT compared with No DVT groups (*P* = 0.008). Furthermore, the NLR and SII levels in DVT group obviously increased compared with No DVT group (*P* < 0.001). (Additional file 2: Table S2). A violin plot was created from the statistically significant data (Fig. 1).

### Factors correlated with DVT among old cases experiencing intertrochanter femoral fracture

**Fig. 2:**
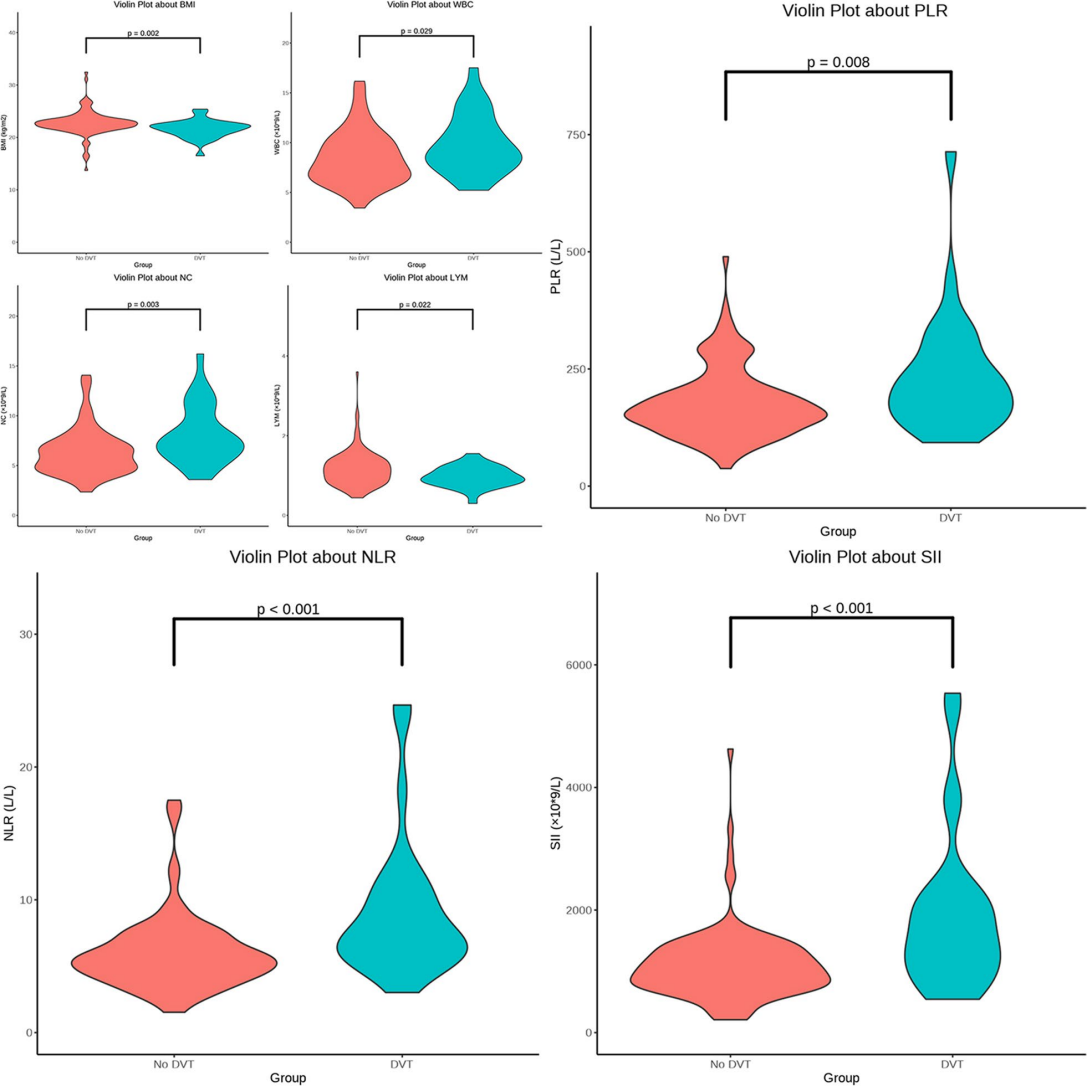
Violin plot of significant variables obtained after univariate logistic regression analysis

**Fig. 3:**
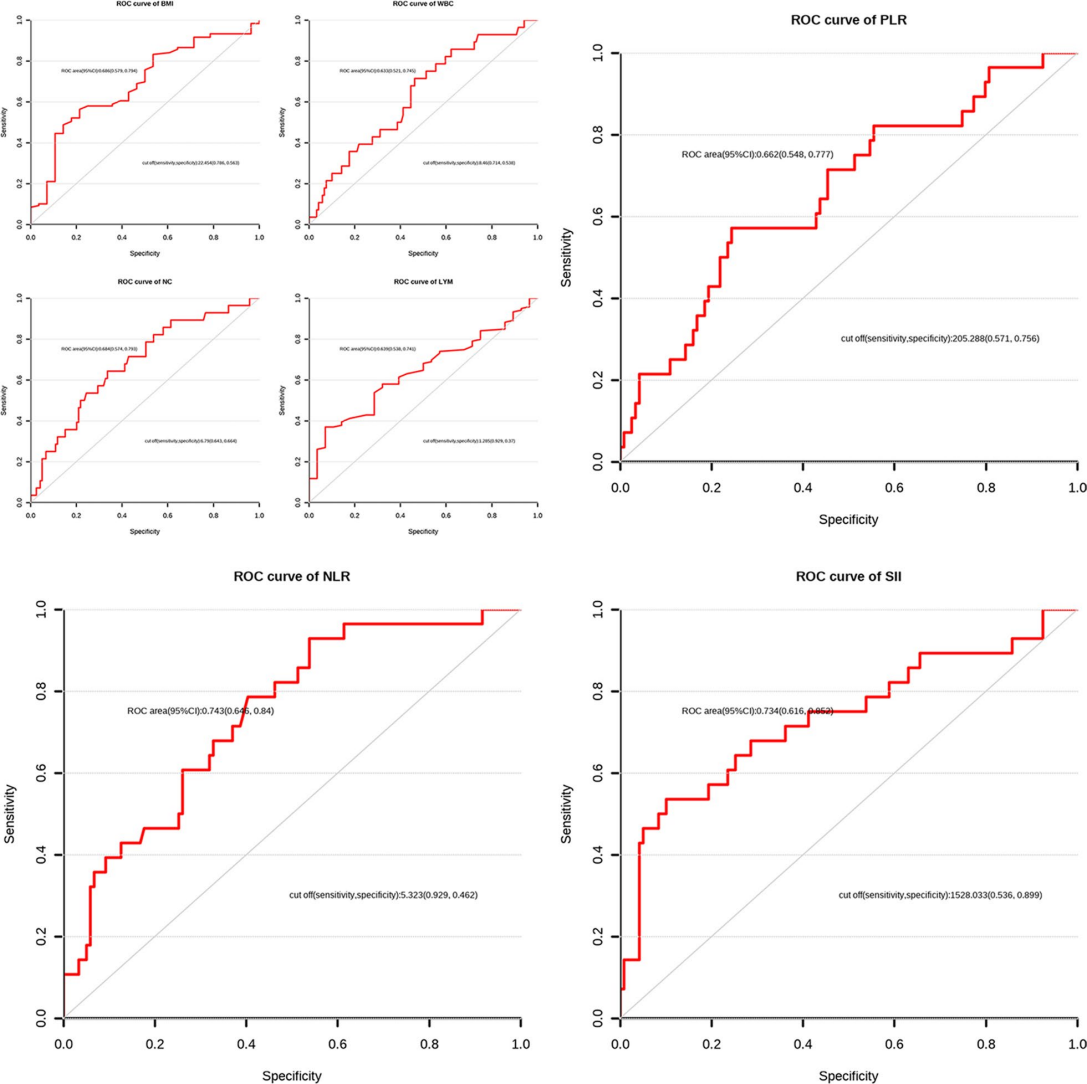
ROC of significant variables obtained after univariate logistic regression analysis

WBC, NC, NLR, PLR, and SII values of DVT group (median values: 8.75 × 10^9^/L, 7.43 × 10^9^/L, 7.05 L/L, 212.69 L/L, and 1558.09 × 10^9^/L, respectively) were notably higher than those in the No DVT group (median values: 8.13 × 10^9^/L, 6.17 × 10^9^/L, 5.46 L/L, 161.18 L/L, and 1013.07 × 10^9^/L, respectively). The value of BMI and LYM in the DVT group (median: 21.84 kg/m^2^, 0.96 × 10^9^/L) obviously decreased compared with No DVT group (median: 22.64 kg/m^2^, 1.12 × 10^9^/L) (P < 0.05, Additional file 2: Table S2, Fig. 2). Based on ROC curves, the optimal thresholds of BMI, WBC, NC, LYM, PLR, and SII were 22.45444 kg/m^2^, 8.46 × 10^9^/L, 6.79 × 10^9^/L, 1.285 × 10^9^/L, 5.323077 L/L, 205.2885 L/L, and 1528.033 L/L, respectively, with a sensitivity of 0.786, 0.714, 0.643, 0.929, 0.929, 0.571, and 0.536, respectively, and specificity of 0.563, 0.538, 0.664, 0.37, 0.462, 0.756, and 0.899, respectively. The AUCs of those data were 0.686, 0.633, 0.684, 0.639, 0.743, 0.662, and 0.734, respectively, which were considered to be a certain predictive value in diagnosing DVT (Additional file 3: Table 3, Fig. 3).

**Fig. 4:**
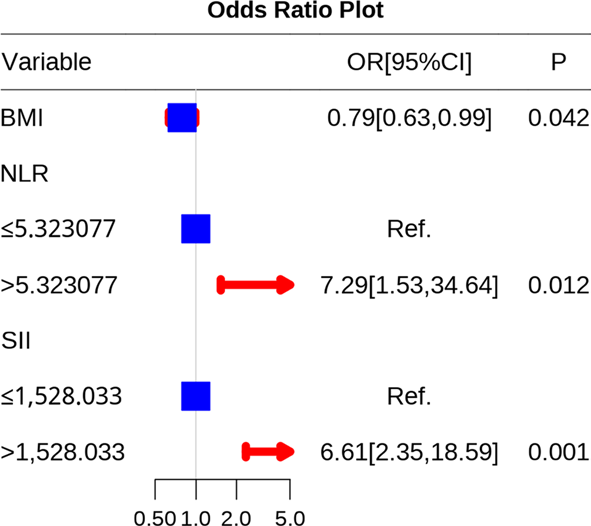
Odds ratio plot of significant variables obtained after multivariate logistic regression analysis

We split the 147 patients into high and low groups on the basis of the ROC cutoff value to better comprehend the relationship between the aforementioned differential variables and DVT. As shown in the univariate analyses, low BMI and high WBC, NC, LYM, NLR, PLR, and SII were linked to DVT. Upon multivariate regression, low BMI, high NLR, and high SII were discovered to be factors independently predicting DVT among cases developing an intertrochanteric femoral fracture (Additional file 4: Table S4, Fig. 4).

### Predictive nomogram development

**Fig. 5:**
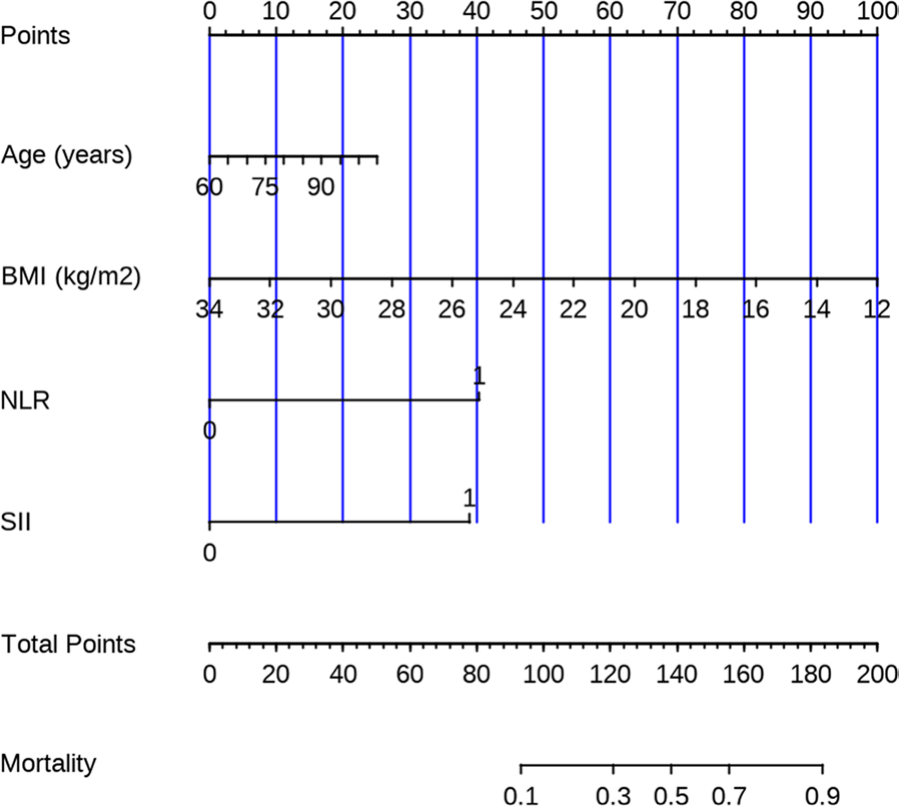
Nomogram of prediction model obtained by binary logistic regression analysis

**Fig. 6:**
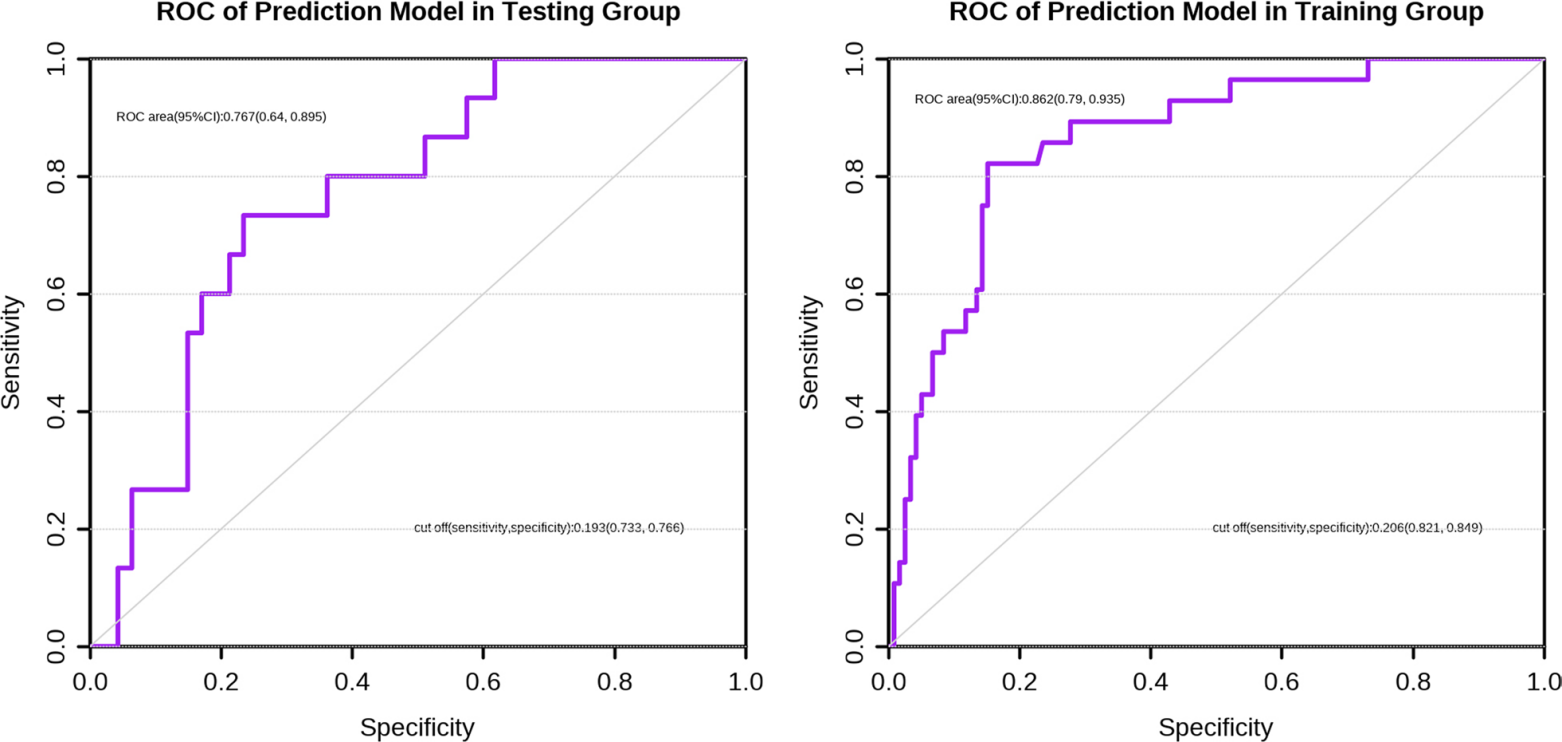
ROC of the prediction in the testing group and the training group

**Fig. 7:**
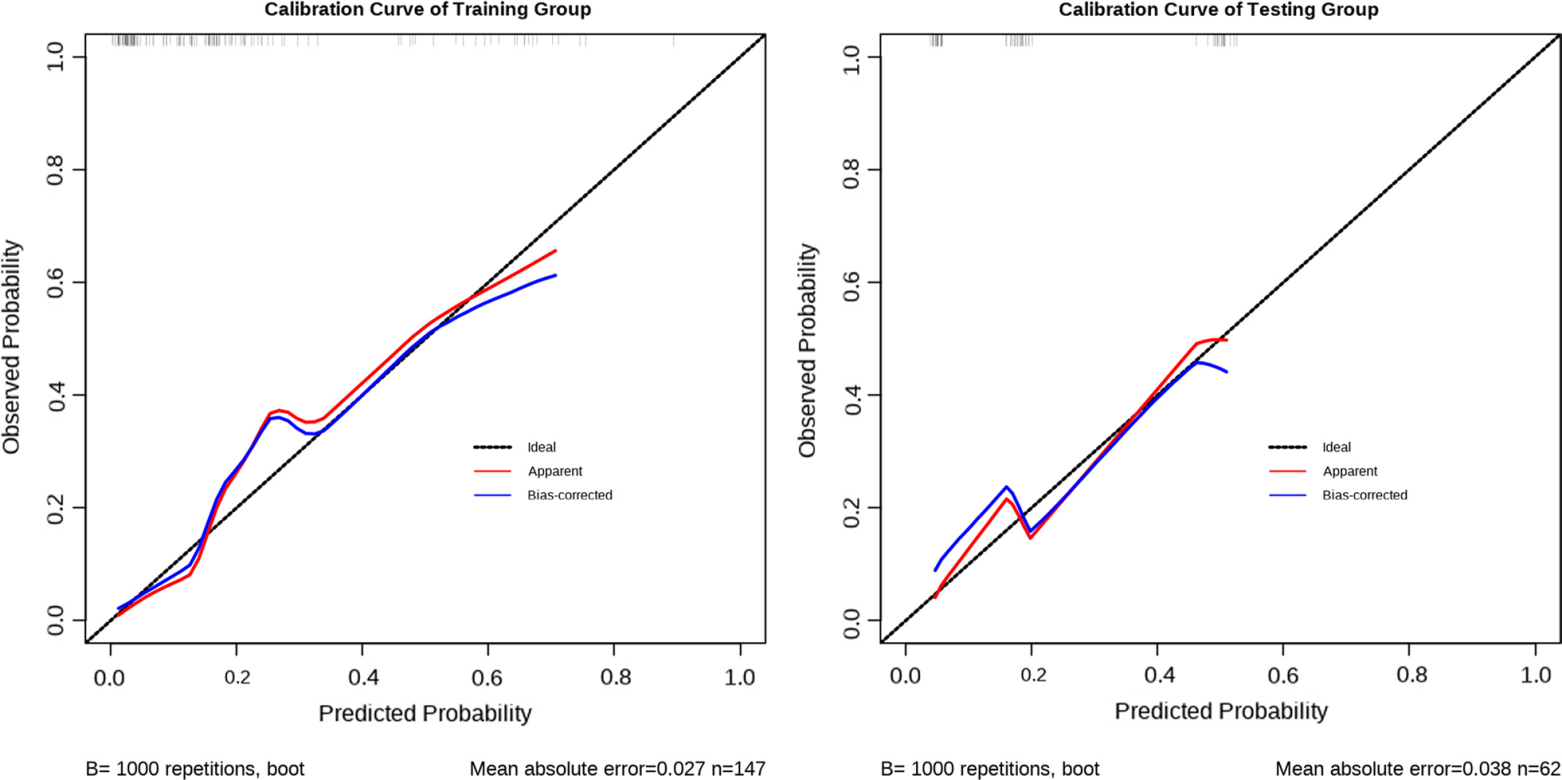
Calibration curve of the training group and the testing group

**Fig. 8:**
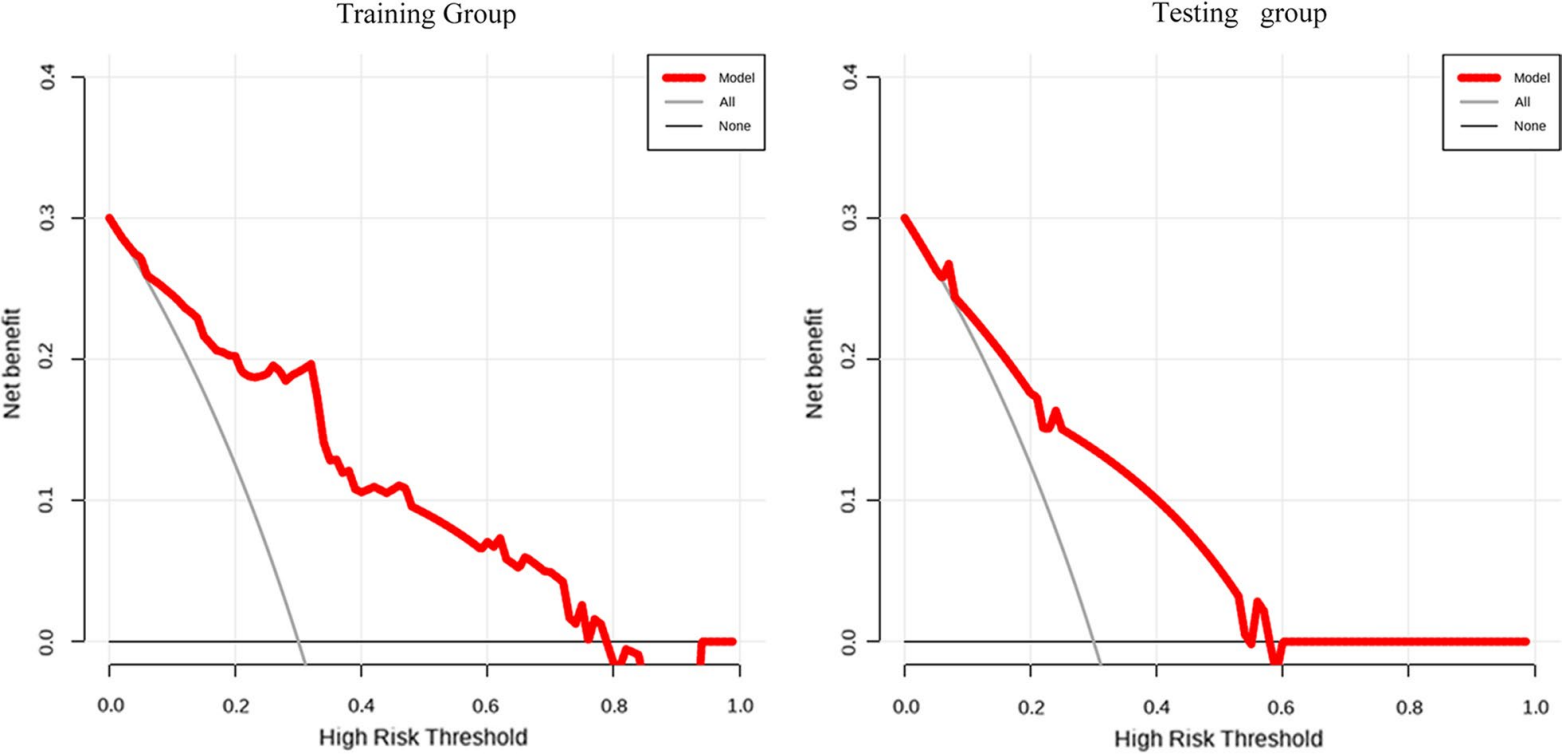
DCA of the training group and the testing group

Next, with the aim of predicting the DVT development among cases developing an intertrochanteric femoral fracture, we created the nomogram model using age, BMI, NLR, and SII using a binary logistic regression equation (Fig. 5). Other indicators, such as NLR and SII, were binary variables, whereas age and BMI were continuous variables. Zero denoted a low NLR, while 1 denoted a high NLR. Similarly, zero denoted a low SII, while 1 stood for a high SII. The calibration curve, nomogram, DCA curve and ROC curve mapped. Our nomogram contained a score for each component, and the aggregate of the values indicated the risk of DVT. The training group's AUC was 0.862 (Fig. 6A), whereas the testing group's AUC was 0.767 (Fig. 6B). The calibration curves were plotted to calibrate our nomogram, that of training group (Fig. 7A) indicated the mean model error of 0.027 for predicting vs. actually producing bad result, while that of testing group (Fig. 7B) was 0.038, indicating that the predictions were conformed to the data. The training group’s DCA curve revealed that when the high-risk threshold was 0.02–0.78, our prediction model could offer superior clinical benefits (Fig. 8A). Testing group’s DCA curve revealed that the prediction model could offer higher clinical benefits when the high-risk threshold was between 0.06 and 0.57 (Fig. 8B).

## Discussion

Intertrochanteric femoral fracture has been a commonly seen fracture that requires hospitalization among the old cases [31]. As global life expectancy and the proportion of older individuals increases, hip fracture risk among the old people, particularly intertrochanteric fractures, will continue to elevate [32]. Survival and prognosis analysis has been the focus of research in recent years. Patients with intertrochanteric fractures often require surgical treatment, and the preoperative and postoperative complications are the major reason for high disability and mortality rates in these patients [33]. DVT is regarded as a frequently seen complication secondary to intertrochanteric fractures [34]. However, the low specificity of a single clinical index and the late completion time of color Doppler ultrasonography often delays the clinical diagnosis of DVT [7–9]. This defers the treatment of DVT, leading to a series of complications, delayed surgery, and even death [3, 12, 35]. In the present study, after multivariate logistic regression analysis, BMI, NLR, and SII were found to be independent predictive factors in the diagnosis of DVT in intertrochanteric femoral fractures. The above prediction indexes are easy to obtain and calculate. For doctors, it can greatly improve the efficiency of diagnosis and treatment of DVT. For patients, it is of great help to reduce the rate of disability and mortality and improve the prognosis. A nomogram prediction model was also constructed based on these three factors, and its validity and practicability in clinical were analyzed. This model can assist in the rapid, accurate, and effective prediction of DVT in patients suffering from intertrochanteric femoral fractures.

Currently, BMI’s effect on DVT pathogenesis is unknown. As per one school of thought, cases showing the high BMI probably show relatively more adipose tissues, which may increase inflammatory factor production and enhance tissue factor generation, in turn activating exogenous coagulation pathways as well as promoting thrombus formation [36]. Matsumoto et al. attempted to analyze age, BMI, and D-dimer in thrombotic cases compared with non-thrombotic cases and showed that BMI was not significantly different between them [37]. Pahlkotter et al. analyzed the ACS-NSQIP database and discovered that the occurrence of DVT in obese patients was 1.7 times that in normal BMI patients, and that in cases having BMI < 18.5 kg/m^2^ was 1.4 folds that in normal BMI patients [38]. This indicates that patients with abnormal BMI, whether obese or thin, have an elevated DVT incidence relative to cases showing normal BMI. As observed from the research on cases receiving total hip replacement, Yu et al. found BMI > 28 kg/m^2^ as the factor predicting DVT incidence [39]. In our study, BMI, as an independent protective factor, was less correlated with DVT than were NLR and SII (OR 0.79; 95% CI 0.63–0.99; *P*, 0.042), which is contrary to the findings of previous studies, possibly due to the narrow range of BMI values in our study (testing group, 22.43 kg/m^2^ (21.62, 23.37); training group, 22.67 kg/m^2^ (21.52, 23.92)). Most patients were roughly within the normal range. This likely led to BMI being a protective factor in the prediction of intertrochanteric fracture thromboses in our model. An increase in BMI within a certain range may represent an increase in muscle strength. The improved blood flow associated with greater muscle strength can reduce the risk of DVT [5]. Nevertheless, our findings need to be confirmed by increasing the sample size or increasing the range of BMI in the population.

Previously, our understanding of DVT was confined to the Virchow triad, but there is now increasing evidence that the immune-inflammatory system also makes a vital impact on the process of thrombosis [40]. Budnik and Brill systematically described the mechanisms of immune factors in thrombosis, such as the depletion of neutrophils that inhibits venous thrombus formation [3]. The process of immune inflammation is described in Branchford's paper. The release of various inflammatory factors under the combined action of neutrophils, T-lymphocytes and circulating monocytes has an important effect on thrombogenesis [14]. Many immune and inflammatory reactions can be detected during DVT occurrence and disappearance [41]. NLR, which includes neutrophils and lymphocytes, collectively represents the immune inflammatory system, and it is also widely used [25, 30, 42]. In this study, NLR was discovered to independently predict thrombosis among cases undergoing intertrochanteric femoral fracture, and had a good predictive value (AUC, 0.743; 95% CI 0.646, 0.84; *P* < 0.001). As easily available clinical data, NLR can enable faster diagnoses and accurate treatments in clinical medicine.

SII is a popular immunoinflammatory index in recent years. It includes the combination of neutrophils, lymphocytes and PLTs, which allows for better prediction in the field of orthopedics. According to our results, SII represented the strong factor predicting DVT among cases experiencing intertrochanteric femoral fractures. When the SII threshold was 1528.033 in the ROC curve, the AUC was 0.734 (95%: 0.616, 0.852; *P* < 0.001), which markedly increased compared with BMI (AUC, 0.686; 95 CI% 0.576, 0.796; *P* < 0.002). Wang et al. used SII for predicting the survival rate of old cases experiencing hip fractures in their prospective cohort study, and concluded that it had a good predictive value and strong clinical practicability [43]. In a multi-arm prospective cohort study, researchers used SII to predict fracture among postmenopausal cases. In this study, age, longer menopause duration, higher NLR, and higher SII were detected to be independent risk factors, among which the predictive effect of SII was more prominent than that of other factors [44]. SII also plays a predictive role in the diagnosis of thrombosis after knee replacement [45]. Therefore, the prediction model constructed in this study, with the combined effect of SII, can help determine the diagnosis and appropriate treatments more quickly and accurately.

However, certain limitations should be noted in this work: (1) This was an unicentric retrospective study which had limited sample size as well as several confounding variables. Clinically significant risk variables, such as time from trauma to admission and age, were not thoroughly examined. A multicenter regression study and thorough examination of data results for risk variables could be conducted in future studies. This will improve the predictive value and accuracy of the model. (2) The preoperative preliminary diagnosis of DVT was caused by a delay in ultrasonography time. When the time to refine the results was extended, the DVT developed in certain individuals differed from the results required for our study. Future studies can incorporate the time of diagnosis, the time of reporting, and the time difference between the two results. (3) This work was not validated by external data and the model's correctness and usefulness were not clarified. When multi-center retrospective studies are conducted in the future, external verification can be carried out with data from other hospitals to confirm the authenticity of the model. (4) A large number of cases were ruled out of this work and the prediction model scope was small. It is suggested to increase the sample size as much as possible on the basis of strictly following the inclusion and exclusion criteria in future multi-center studies. (5) This study only studied the risk of DVT before surgery. The further discussion on the risk of DVT after surgery can be carried out in the future.

## Conclusion

The BMI, NLR, and SII at admission are independent predictors of DVT in patients with intertrochanteric femoral fractures. These indices can be acquired conveniently and quickly. In addition, the nomogram on the basis of BMI, NLR, and SII can assist clinicians in evaluating the need for DVT. In clinical practice, faster and better treatment decisions can be made visually.

## Supplementary Information

**Additional file 1**. **Table S1**: Baseline characteristics of all studied patients.

**Additional file 2**. **Table S2**: Comparison of clinical and laboratory data between the No DVT group and DVT group in the Testing Group.

**Additional file 3**. **Table S3**: AUC and Cutoff value of ROC curve in the significant variables.

**Additional file 4**. **Table S4**: Outcomes of the binary logistic regression analysis.

## Data Availability

All data supporting the present work are contained in this study, which can be obtained from corresponding authors on request.

## Abbreviations

DVT: Deep vein thrombosis
BMI: Body mass index
FIB: Fibrinogen
PT: Prothrombin time
TT: Thrombin time
PT–INR: Prothrombin time–international normalized ratio
APTT: Activated partial thromboplastin time
PLT: Platelet count
MNC: Mononuclear cell
NC: Neutrophilic cell
LYM: Lymphocyte
WBC: White blood cell
RBC: RED blood cell
HCT: Red blood cell specific volume
MCV: Mean corpuscular volume
MCH: Mean corpuscular hemoglobin
MCHC: Mean corpuscular hemoglobin concentration
HGB: Hemoglobin
PDW: Platelet distributionwidth
FBG: Fasting blood glucose
DBP: Diastolic blood pressure
SBP: Systolic blood pressure
Dtime: Damage time
MLR: Monocyte/lymphocyte ratio
PLR: Platelet/lymphocyte ratio
SII: Systemic immune-inflammation index
NLR: Neutrophil/lymphocyte ratio, platelet × neutrophilic cell/lymphocyte
DCA: Decision curve analysis
AUC: Area under the curve
ROC: Receiver operating characteristic curve
ORs: Odds ratios
CIs: Confidence intervals
