# Supplementary material for: A nomogram model based on the combination of the systemic immune-inflammation index, body mass index, and neutrophil/lymphocyte ratio to predict the risk of preoperative deep venous thrombosis in elderly patients with intertrochanteric femoral fracture: a retrospective cohort study
Source: J Orthop Surg Res. 2023 Aug 3;18:561. doi: 10.1186/s13018-023-03966-4 (PMC10398922; doi:10.1186/s13018-023-03966-4)
Supplement: Supplementary file 1 — Additional file 1. Table S1: Baseline characteristics of all studied patients. [file 13018_2023_3966_MOESM1_ESM.docx]

**Table S1.** Baseline characteristics of all studied patients

| **Variable** |  | **Data groups** | |  |  |
| --- | --- | --- | --- | --- | --- |
|  | Total  (n = 209) | Training group  (n = 147) | Testing group  (n = 62) | Statistics | P |
| **Sex (n)** |  |  |  | 0 | 0.995 |
| **man** | 54(25.84%) | 38(25.85%) | 16(25.81%) |  |  |
| **woman** | 155(74.16%) | 109(74.15%) | 46(74.19%) |  |  |
| **Age (years)** | 83(77, 87) | 83(77, 87) | 83.5(74.5, 87) | 0.063 | 0.950 |
| **BMI (kg/m2)** | 22.48(21.62, 23.5) | 22.43(21.62, 23.37) | 22.67(21.52, 23.92) | -0.934 | 0.350 |
| **Alcoholism (n)** |  |  |  | 0.673 | 0.412 |
| **yes** | 30(14.35%) | 23(15.65%) | 7(11.29%) |  |  |
| **no** | 179(85.65%) | 124(84.35%) | 55(88.71%) |  |  |
| **Smoke (n)** |  |  |  | 0.402 | 0.526 |
| **yes** | 48(22.97%) | 32(21.77%) | 16(25.81%) |  |  |
| **no** | 161(77.03%) | 115(78.23%) | 46(74.19%) |  |  |
| **Hypertension (n)** |  |  |  | 0.011 | 0.918 |
| **yes** | 63(30.14%) | 44(29.93%) | 19(30.65%) |  |  |
| **no** | 146(69.86%) | 103(70.07%) | 43(69.35%) |  |  |
| **Diabetes (n)** |  |  |  | 0.337 | 0.561 |
| **yes** | 28(13.4%) | 21(14.29%) | 7(11.29%) |  |  |
| **no** | 181(86.6%) | 126(85.71%) | 55(88.71%) |  |  |
| **Fracture site (n)** |  |  |  | 0.375 | 0.54 |
| **left** | 118(56.46%) | 85(57.82%) | 33(53.23%) |  |  |
| **right** | 91(43.54%) | 62(42.18%) | 29(46.77%) |  |  |
| **Thrombus (n)** |  |  |  | 0.707 | 0.401 |
| **no** | 166(79.43%) | 119(80.95%) | 47(75.81%) |  |  |
| **yes** | 43(20.57%) | 28(19.05%) | 15(24.19%) |  |  |
| **SBP (mmHg)** | 137.61 ± 23.05 | 138.29 ± 23.97 | 136 ± 20.78 | 0.654 | 0.514 |
| **DBP (mmHg)** | 77(67,85) | 76(65, 84.5) | 78(70, 84.75) | -1.021 | 0.307 |
| **Dtime (hours)** | 24(7,25) | 24(5.5, 24) | 24(10.25, 25.75) | -0.771 | 0.440 |
| **Temperature (℃)** | 36.6(36.5, 36.7) | 36.6(36.5, 36.7) | 36.6(36.5, 36.7) | 1.349 | 0.177 |
| **BPM (times/minute)** | 20(19, 20) | 20(19, 20) | 20(19.25, 20) | -1.028 | 0.304 |
| **Pulse (times/minute)** | 85.21 ± 13.11 | 84.86 ± 13.44 | 86.02 ± 12.35 | -0.579 | 0.563 |

**** SBP*** Systolic Blood pressure, ***DBP*** Diastolic Blood pressure, ***Dtime*** Damage time, ***BPM*** Breaths Per Minute
