# Supplementary material for: A nomogram model based on the combination of the systemic immune-inflammation index, body mass index, and neutrophil/lymphocyte ratio to predict the risk of preoperative deep venous thrombosis in elderly patients with intertrochanteric femoral fracture: a retrospective cohort study
Source: J Orthop Surg Res. 2023 Aug 3;18:561. doi: 10.1186/s13018-023-03966-4 (PMC10398922; doi:10.1186/s13018-023-03966-4)
Supplement: Supplementary file 2 — Additional file 2. Table S2: Comparison of clinical and laboratory data between the No DVT group and DVT group in the Testing Group. [file 13018_2023_3966_MOESM2_ESM.docx]

**Table S2.** Comparison of clinical and laboratory data between the No DVT group and DVT group in the Testing Group

| **Variable** |  |  |  | **Testing Group** | | | |  |  |
| --- | --- | --- | --- | --- | --- | --- | --- | --- | --- |
|  |  | Patients  (n) | Total | Patients (n) | No DVT group | Patients (n) | DVT group | Statistic | P |
| **Fracture site (n)** |  |  |  |  |  |  |  | 0.592 | 0.442 |
|  | Left |  | 85(57.82%) |  | 67(56.3%) |  | 18(64.29%) |  |  |
|  | Right |  | 62(42.18%) |  | 52(43.7%) |  | 10(35.71%) |  |  |
| **Hypertension (n)** |  |  |  |  |  |  |  | 0.551 | 0.458 |
|  | yes |  | 44(29.93%) |  | 34(28.57%) |  | 10(35.71%) |  |  |
|  | no |  | 103(70.07%) |  | 85(71.43%) |  | 18(64.29%) |  |  |
| **Diabetes (n)** |  |  |  |  |  |  |  | 0 | 1 |
|  | yes |  | 21(14.29%) |  | 17(14.29%) |  | 4(14.29%) |  |  |
|  | no |  | 126(85.71%) |  | 102(85.71%) |  | 24(85.71%) |  |  |
| **Sex (n)** |  |  |  |  |  |  |  | 0.013 | 0.909 |
|  | man |  | 38(25.85%) |  | 31(26.05%) |  | 7(25%) |  |  |
|  | woman |  | 109(74.15%) |  | 88(73.95%) |  | 21(75%) |  |  |
| **Age (years)** |  | 147 | 83(77, 87) | 119 | 83(75.5, 87) | 28 | 85.5(82, 87.25) | -1.343 | 0.179 |
| **BMI (kg/m2)** |  | 147 | 22.43  (21.62, 23.37) | 119 | 22.64  (21.84, 23.44) | 28 | 21.84  (20.7, 22.41) | 3.064 | **0.002** |
| **Smoke (n)** |  |  |  |  |  |  |  | 0.311 | 0.577 |
|  | yes |  | 32(21.77%) |  | 27(22.69%) |  | 5(17.86%) |  |  |
|  | no |  | 115(78.23%) |  | 92(77.31%) |  | 23(82.14%) |  |  |
| **Alcoholism (n)** |  |  |  |  |  |  |  | 0 | 1 |
|  | yes |  | 23(15.65%) |  | 19(15.97%) |  | 4(14.29%) |  |  |
|  | no |  | 124(84.35%) |  | 100(84.03%) |  | 24(85.71%) |  |  |
| **SBP (mmHg)** |  | 147 | 138.29 ± 23.97 | 119 | 138.87 ± 22.95 | 28 | 135.82 ± 28.25 | 0.603 | 0.547 |
| **DBP (mmHg)** |  | 147 | 76(65, 84.5) | 119 | 76(65.5, 85) | 28 | 75.5(65, 84) | 0.257 | 0.797 |
| **Dtime (hours)** |  | 147 | 24(5.5, 24) | 119 | 24(5.5, 24) | 28 | 22(5.5, 26.75) | -0.126 | 0.9 |
| **Temperature (℃)** |  | 147 | 36.6  (36.5, 36.7) | 119 | 36.6  (36.5, 36.7) | 28 | 36.7  (36.5, 36.8) | -1.197 | 0.231 |
| **BPM (times/minute)** |  | 147 | 20(19, 20) | 119 | 20(19, 20) | 28 | 20(20, 20) | -1.288 | 0.198 |
| **Pulse (times/minute)** |  | 147 | 84.86 ± 13.44 | 119 | 84.47 ± 13.4 | 28 | 86.54 ± 13.74 | -0.730 | 0.466 |
| **HGB (g/L)** |  | 147 | 99.83 ± 19.56 | 119 | 99.05 ± 19.7 | 28 | 103.14±18.94 | -0.996 | 0.321 |
| **RBC (×10^12^/L)** |  | 147 | 3.44  (2.98, 3.91) | 119 | 3.46  (2.98, 3.88) | 28 | 3.25  (3.01, 4.03) | 0.143 | 0.886 |
| **WBC (×10^9^/L)** |  | 147 | 8.49  (6.63, 10.39) | 119 | 8.13(6.5, 10.16) | 28 | 8.75  (7.97, 11.43) | -2.185 | **0.029** |
| **NC (×10^9^/L)** |  | 147 | 6.36  (4.88, 7.7) | 119 | 6.17  (4.72, 7.3) | 28 | 7.43  (6.06, 9.34) | -3.019 | **0.003** |
| **LYM (×10^9^/L)** |  | 147 | 1.1  (0.85, 1.33) | 119 | 1.12  (0.86, 1.4) | 28 | 0.96  (0.81, 1.19) | 2.289 | **0.022** |
| **MNC (×10^9^/L)** |  | 147 | 0.61  (0.5, 0.82) | 119 | 0.61  (0.5, 0.82) | 28 | 0.59  (0.48, 0.83) | 0.533 | 0.594 |
| **NLR (L/L)** |  | 147 | 5.75  (4.6, 7.46) | 119 | 5.46  (4.37, 7.09) | 28 | 7.05  (5.95, 10.6) | -3.993 | **<0.001** |
| **PLR (L/L)** |  | 147 | 167.27  (136.74, 217.98) | 119 | 161.18  (131.57, 202.08) | 28 | 212.69  (157.75, 270.23) | -2.664 | **0.008** |
| **MLR (L/L)** |  | 147 | 0.57(0.42, 0.77) | 119 | 0.57(0.4, 0.75) | 28 | 0.66(0.45, 0.81) | -1.359 | 0.174 |
| **PLT (×10^9^/L)** |  | 147 | 191  (155, 223) | 119 | 189  (152.5, 220) | 28 | 202  (167.75, 229.25) | -1.211 | 0.226 |
| **SII (×10^9^/L)** |  | 147 | 1081.53(793.17, 1430.03) | 119 | 1013.07(760.17, 1320.29) | 28 | 1558.09(1091.03, 2114.79) | -3.848 | **<0.001** |
| **FIB (g/L)** |  | 146 | 3.5(2.9,4.3) | 118 | 3.5(2.9, 4.26) | 28 | 3.4(2.82, 4.7) | -0.065 | 0.948 |
| **TT (s)** |  | 146 | 16.5  (16,17.67) | 118 | 16.5  (15.9,17.7) | 28 | 16.4  (16.08,17.45) | 0.065 | 0.948 |
| **PT-INR** |  | 146 | 0.98  (0.94,1.03) | 118 | 0.98  (0.94,1.03) | 28 | 0.98  (0.94,1.02) | 0.368 | 0.713 |
| **APTT (s)** |  | 146 | 27.35  (24.33,32.18) | 118 | 27.65(24.45,32.1) | 28 | 25.7  (23.92,32.25) | 0.887 | 0.375 |
| **D-dimmer (mg/L)** |  | 142 | 8.14(4.12,9.4) | 116 | 7.75(4.24,9.4) | 26 | 9.4(2.87,9.4) | -0.688 | 0.491 |
| **PT (s)** |  | 146 | 11.4  (10.9,11.88) | 118 | 11.35  (10.9,11.8) | 28 | 11.4  (10.95,11.9) | 0.137 | 0.891 |

*** ***SBP*** Systolic Blood Pressure, ***DBP*** Diastolic Blood pressure, ***Dtime*** Damage time, ***BPM*** Breaths Per Minute, ***HGB*** Hemoglobin, ***RBC*** Red Blood Cell, ***WBC*** White Blood Cell, ***NC*** Neutrophilic cell, ***LYM*** lymphocyte, ***MNC*** mononuclear cells, ***PLT*** Platelet, ***FIB*** Fibrinogen, ***TT*** Thrombintime, ***APTT*** Activated Partial Thromboplastin Time, ***PT*** Prothrombin Time

*******NLR*** NC/LYM, ***MLR*** MNC/LYM, ***PLR*** PLT/LYM, ***SII*** NE*PLT/LYM
