# Supplementary material for: A nomogram model based on the combination of the systemic immune-inflammation index, body mass index, and neutrophil/lymphocyte ratio to predict the risk of preoperative deep venous thrombosis in elderly patients with intertrochanteric femoral fracture: a retrospective cohort study
Source: J Orthop Surg Res. 2023 Aug 3;18:561. doi: 10.1186/s13018-023-03966-4 (PMC10398922; doi:10.1186/s13018-023-03966-4)
Supplement: Supplementary file 3 — Additional file 3. Table S3: AUC and Cutoff value of ROC curve in the significant variables. [file 13018_2023_3966_MOESM3_ESM.docx]

**Table S3.** AUC and Cutoff value of ROC curve in the significant variables

| **Variable** | **Patients** | **AUC** | **SE** | **P** | **AUC [95%CI]** | **Cutoff value** | **Sensitivity** | **Specificity** | **Accuracy** |
| --- | --- | --- | --- | --- | --- | --- | --- | --- | --- |
| **BMI** | 147 | 0.686 | 0.056 | **0.002** | [0.576,0.796] | 22.45444 | 0.786 | 0.563 | 0.605 |
| **WBC** | 147 | 0.633 | 0.055 | **0.029** | [0.525,0.741] | 8.46 | 0.714 | 0.538 | 0.571 |
| **NC** | 147 | 0.684 | 0.055 | **0.003** | [0.576,0.792] | 6.79 | 0.643 | 0.664 | 0.66 |
| **LYM** | 147 | 0.639 | 0.051 | **0.022** | [0.539,0.739] | 1.285 | 0.929 | 0.37 | 0.476 |
| **NLR** | 147 | **0.743** | 0.048 | **<0.001** | [0.649,0.837] | 5.323077 | 0.929 | 0.462 | 0.551 |
| **PLR** | 147 | 0.662 | 0.057 | **0.008** | [0.55,0.774] | 205.2885 | 0.571 | 0.756 | 0.721 |
| **SII** | 147 | **0.734** | 0.06 | **<0.001** | [0.616,0.852] | 1,528.033 | 0.536 | 0.899 | 0.83 |
