# Supplementary material for: A nomogram model based on the combination of the systemic immune-inflammation index, body mass index, and neutrophil/lymphocyte ratio to predict the risk of preoperative deep venous thrombosis in elderly patients with intertrochanteric femoral fracture: a retrospective cohort study
Source: J Orthop Surg Res. 2023 Aug 3;18:561. doi: 10.1186/s13018-023-03966-4 (PMC10398922; doi:10.1186/s13018-023-03966-4)
Supplement: Supplementary file 4 — Additional file 4. Table S4: Outcomes of the binary logistic regression analysis. [file 13018_2023_3966_MOESM4_ESM.docx]

### Table S4. Outcomes of the binary logistic regression analysis

| **Variable** |  | **Univariate analysis** | | | **Multivariate analysis** | |
| --- | --- | --- | --- | --- | --- | --- |
|  |  | Patients | OR [95%CI] | Log-rank P | OR [95%CI] | Log-rank P |
| **BMI (kg/m2)** |  | 147 |  | **0.002** | 0.79[0.63,0.99] | **0.042** |
|  | ≤22.45444 | 74(50.34%) | 1 |  |  |  |
|  | >22.45444 | 73(49.66%) | 0.21[0.08,0.56] |  |  |  |
| **WBC (×10^9^/L)** |  | 147 |  | **0.019** |  |  |
|  | ≤8.46 | 72(48.98%) | 1 |  |  |  |
|  | >8.46 | 75(51.02%) | 3[1.11,8.09] |  |  |  |
| **NC (×10^9^/L)** |  | 147 |  | **0.004** |  |  |
|  | ≤6.79 | 89(60.54%) | 1 |  |  |  |
|  | >6.79 | 58(39.46%) | 2.91[1.19,7.12] |  |  |  |
| **LYM (×10^9^/L)** |  | 147 |  | **0.007** |  |  |
|  | ≤1.285 | 101(68.71%) | 1 |  |  |  |
|  | >1.285 | 46(31.29%) | 3.55[1.5,8.41] |  |  |  |
| **NLR (L/L)** |  | 147 |  | **<0.001** | 7.29[1.53,34.64] | **0.012** |
|  | ≤5.323077 | 102(69.39%) | 1 |  |  |  |
|  | >5.323077 | 45(30.61%) | 11.17[2.54,49.21] |  |  |  |
| **PLR (L/L)** |  | 147 |  | **0.001** |  |  |
|  | ≤205.2885 | 87(59.18%) | 1 |  |  |  |
|  | >205.2885 | 60(40.82%) | 4.14[1.76,9.75] |  |  |  |
| **SII (×10^9^/L)** |  | 147 |  | **<0.001** | 6.61[2.35,18.59] | **<0.001** |
|  | ≤1,528.033 | 120(81.63%) | 1 |  |  |  |
|  | >1,528.033 | 27(18.37%) | 10.29[3.97,26.67] |  |  |  |
